# Progressive Remote Axonal Degeneration Following Spinal Cord Injury: A Histological and MRI Study

**DOI:** 10.1089/neur.2025.0011

**Published:** 2025-06-05

**Authors:** Gergely David, Alice Motovylyak, Felix Schlegel, Zsofia Kovacs, Christian Kündig, Angela R. Filous, Jan M. Schwab, Matthew D. Budde, Jan Klohs, Patrick Freund

**Affiliations:** ^1^ Spinal Cord Injury Center, Balgrist University Hospital, University of Zurich, Zurich, Switzerland.; ^2^ Department of Biomedical Engineering, Marquette University and Medical College of Wisconsin, Milwaukee, Wisconsin, USA.; ^3^ Institute for Biomedical Engineering, University of Zurich and ETH Zurich, Zurich, Switzerland.; ^4^ Belford Center for Spinal Cord Injury, The Ohio State University, Columbus, Ohio, USA.; ^5^ Department of Neurology, The Ohio State University, Wexner Medical Center, Columbus, Ohio, USA.; ^6^ Center for Brain and Spinal Cord Repair, The Ohio State University, Columbus, Ohio, USA.; ^7^ Department of Neuroscience, The Ohio State University, Columbus, Ohio, USA.; ^8^ Department of Neurosurgery, Clement J Zablocki Veterans Affairs Medical Center, Medical College of Wisconsin, Milwaukee, Wisconsin, USA.; ^9^ Neuroscience Center Zurich, University of Zurich and ETH Zurich, Zurich, Switzerland.; ^10^ Department of Neurophysics, Max Planck Institute for Human Cognitive and Brain Sciences, Leipzig, Germany.; ^11^ Wellcome Trust Centre for Human Neuroimaging, UCL Queen Square Institute of Neurology, University College London, London, United Kingdom.

**Keywords:** atrophy, axonal degeneration, diffusion tensor imaging, immunohistochemistry, remote degeneration, traumatic spinal cord injury

## Abstract

In acute human spinal cord injury (SCI), magnetic resonance imaging (MRI) reveals progressive neuroanatomical changes at the lesion site and in remote regions. Here, we aimed to elucidate the structural underpinnings of these neuroanatomical changes and to characterize their spatiotemporal distribution in a rat contusion SCI model, using both histology and MRI. First, rats subjected to a thoracic contusion SCI (T8) and sham-operated rats were sacrificed at 56 days post-injury (dpi), and SMI-32 immunohistochemistry was used to assess remote axonal degeneration at cervical segments C2–C5. Second, to evaluate the effect of severity and time since injury on axonal degeneration, rats of varying injury severity were sacrificed at 2, 30, and 90 dpi, respectively, followed by SMI-32 immunohistochemistry. Third, *ex vivo* structural MRI and diffusion tensor imaging were performed rostral to the injury site (C3–T6) at 90 dpi. Histological evidence of axonal degeneration emerged as early as 2 dpi rostral to the injury site, persisting at 90 dpi. Severity-dependent degeneration occurred within the fasciculus gracilis and the periphery of the medio- and ventrolateral columns. Corresponding MRI changes, including lower fractional anisotropy in these regions and smaller gray matter area, were detected. In contrast, the dorsal corticospinal tract exhibited lower fractional anisotropy without clear histological abnormalities, potentially due to atrophy-related mislocalization. This highlights the value of correlative, multimodal approaches and the need for further methodological refinement. The number of SMI-32+ axonal profiles correlated negatively, while gray matter area and fractional anisotropy correlated positively with locomotion assessed by Basso, Beattie, and Bresnahan scores. This study demonstrates in independent experiments that neuroanatomical MRI changes observed after SCI, occurring remote from the injury site, are linked to axonal degeneration. Experimental SCI offers translational insights into underlying mechanisms and potential avenues for neuroprotective or rehabilitative approaches.

## Introduction 

Traumatic spinal cord injury (SCI) results from an immediate insult to the spinal cord, triggering pathological changes at the injury site, such as necrosis, apoptosis, edema, hemorrhage, and inflammation.^1^ The damage, however, extends beyond the injury epicenter, spreading both rostrally and caudally along the neuraxis via anterograde (Wallerian)^2–4^ and retrograde degeneration.^5^ Progressive breakdown of myelin and axons, along with clearance of cellular debris through phagocytosis, culminates in atrophy.^6^

While experimental SCI studies mainly focus on the injury epicenter and adjacent regions,^7–10^ there has been limited attention to neurodegenerative processes occurring at distal spinal cord segments.^11^ In clinical studies, the imaging of the spinal segments remote to the lesion site is appealing, as the spinal cord at the injury site is frequently obscured by artifacts arising from spinal instrumentation.^12,13^ However, these studies have not directly examined the pathological features of these remote neuroanatomical changes.

This study aims to better understand remote above-lesion axonal degeneration in a rat thoracic contusion SCI model using a combined histology and magnetic resonance imaging (MRI) approach. We first investigated the spatial distribution of above-lesion axonal degeneration in the upper cervical cord at 56 days post-injury (dpi) using SMI-32 immunohistochemistry (Experiment 1). In a separate cohort, we assessed the temporal dynamics of axonal degeneration between 2 and 90 dpi and how the degenerative changes relate to the severity of SCI (Experiment 2). In a third cohort, we investigated corresponding macro- and microstructural changes at 90 dpi by applying *ex vivo* structural MRI and diffusion tensor imaging (DTI) (Experiment 3). Finally, we characterized the relationship between histology, MRI metrics, and locomotion (Experiments 2 and 3).

## Materials and Methods

**FIG. 1.**
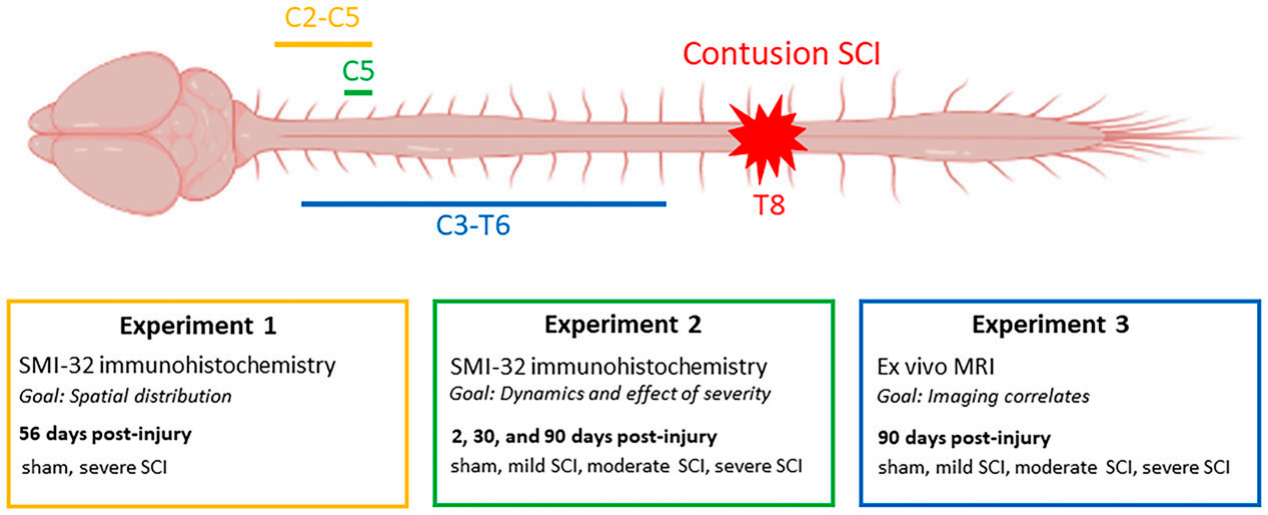
Overview of the experimental design. The overarching objective is the investigation of the remote above-lesion axonal degeneration after contusion SCI, where each experiment focuses on a different aspect: Experiment 1 examines the spatial distribution of degeneration, Experiment 2 investigates its dynamics and the impact of injury severity, and Experiment 3 focuses on imaging correlates by employing *ex vivo* MRI. Part of the figure was created with BioRender.com. SCI, spinal cord injury.

Three distinct experiments were done to investigate various aspects of remote above-level axonal degeneration in a contusion SCI rat model (Fig. 1).

### Experiment 1: Investigating the spatial distribution of remote above-lesion axonal degeneration using SMI-32 immunohistochemistry

#### Contusion SCI rat model

The experiment involved nine male Sprague Dawley rats (Envigo Laboratories), weighing 200–250 g, that underwent either contusion SCI (*n =* 5) or sham surgery (*n =* 4). Rats were anesthetized with a cocktail of ketamine (80 mg/kg in sterile water) and xylazine (10 mg/kg in sterile water) intraperitoneally, and supplemented as needed. Aseptic techniques were followed in accordance with Ohio State University Institutional Animal Care and Use Committee (OSU IACUC) survival surgery guidelines. Once a surgical level of anesthesia was confirmed, the surgery site was prepared with hair removal and sterile preparation of the surgical site. Gentamycin (5 mg/kg, subcutaneous) was administered prior to surgery to prevent confounding infections. The rats were placed on a warming pad to maintain body temperature. A single-level laminectomy at thoracic vertebral level T8, approximately corresponding to spinal level T10, was performed. The rat was placed on a stereotaxic platform, with forceps on T7 and T9 to stabilize the spinal cord for contusion. A contusion SCI was delivered to T8 using IH-0400 Infinite Horizon impactor (PSI, Lexington) with an impact force of 250 kdyne to induce severe injury. The muscles were sutured with 4-0 Demalon suture, and the skin was closed with wound clips. To prevent dehydration after surgery, all rats received 5 cc of 0.9% sterile saline subcutaneously and were placed on a cage warmer overnight for recovery. Rats were placed on post-operative care, including twice-daily bladder expression and a once-daily dose of gentamicin (5 mg/kg subcutaneously, for the first 5 days postoperatively), and sterile saline (5 mL on days 1 and 2, then 4, 3, and 2 mL for the next 3 days, respectively). Animal weights and urine pH were obtained weekly to monitor for signs of distress or infection. Animals were euthanized at 56 dpi. All animal procedures were approved by the Institutional Animal Care and Use Committees at The Ohio State University Wexner Medical Center.

#### SMI-32 immunohistochemistry

Animals were given a lethal dose of ketamine and xylazine and perfused by inserting a blunt needle attached to a perfusion pump into the left ventricle. After an initial flush with phosphate-buffered saline, the fixative was switched to 4% paraformaldehyde. The excised spinal cord was fixed in a paraformaldehyde solution for 24 h and then switched into a phosphate-buffered saline solution (for paraffin-embedded tissue) until further processing. The paraffin-embedded tissue was sectioned to a thickness of 5 µm. After a deparaffinization/hydration sequence, sections were subject to heat induced antigen retrieval with a sodium citrate solution (2.94 g sodium citrate dihydrate, 1 L dH_2_O, adjusted to a pH of 6). The sections were treated with a secondary specific normal block (phosphate-buffered saline (1×), Tx-100 (0.3%), Horse Serum (5%)) for 1 h at room temperature (RT) and then incubated overnight at 4°C with the primary antibody against nonphosphorylated neurofilament (SMI-32; 1:7500; Biolegend 801701). After washing with phosphate-buffered saline, a horse anti-mouse secondary antibody was applied for 1 h at RT (Biot. anti-mouse; 1:2000; Vector BA-2000). After incubation, endogenous peroxidase was inhibited with 6% H_2_O_2_ in methanol for 20 min at RT. The expression was amplified using Elite ABC (Vectastain, PK-6100) for 40 min at RT. The antibody complex was visualized using the ImmPACT DAB HRP Kit (Vector SK 4105) and counterstained with Mayer’s Hematoxylin for 2 min (Sigma-Aldrich; MHs1). After a dH_2_O wash, slides were dehydrated and cover-slipped using permount mounting medium (Fisher Chemical SP15100).

The analyzed regions were localized more than 2 cm rostral to the T8 lesion core, corresponding to spinal levels C2–C5 (Fig. 3A). In each animal, a single representative 5-µm section was imaged using a Nikon Eclipse Ni microscope and NIS-Elements BR 5.02.01 64-bit software. Images were captured at 10x magnification and montaged to generate an image of the entire tissue section (Fig. 3B, C). SMI-32+ axonal profiles were counted in five nonoverlapping high-power fields (HPFs) at 400× magnification (Fig. 3D) within each region (dorsal and dorso-/mediolateral WM), with an eyepiece grid representing 0.0625 mm^2^. Similar to Beschorner et al.,^14^ the selected HPFs were confined to areas with the highest density of labeled axonal profiles.

**FIG. 2.**
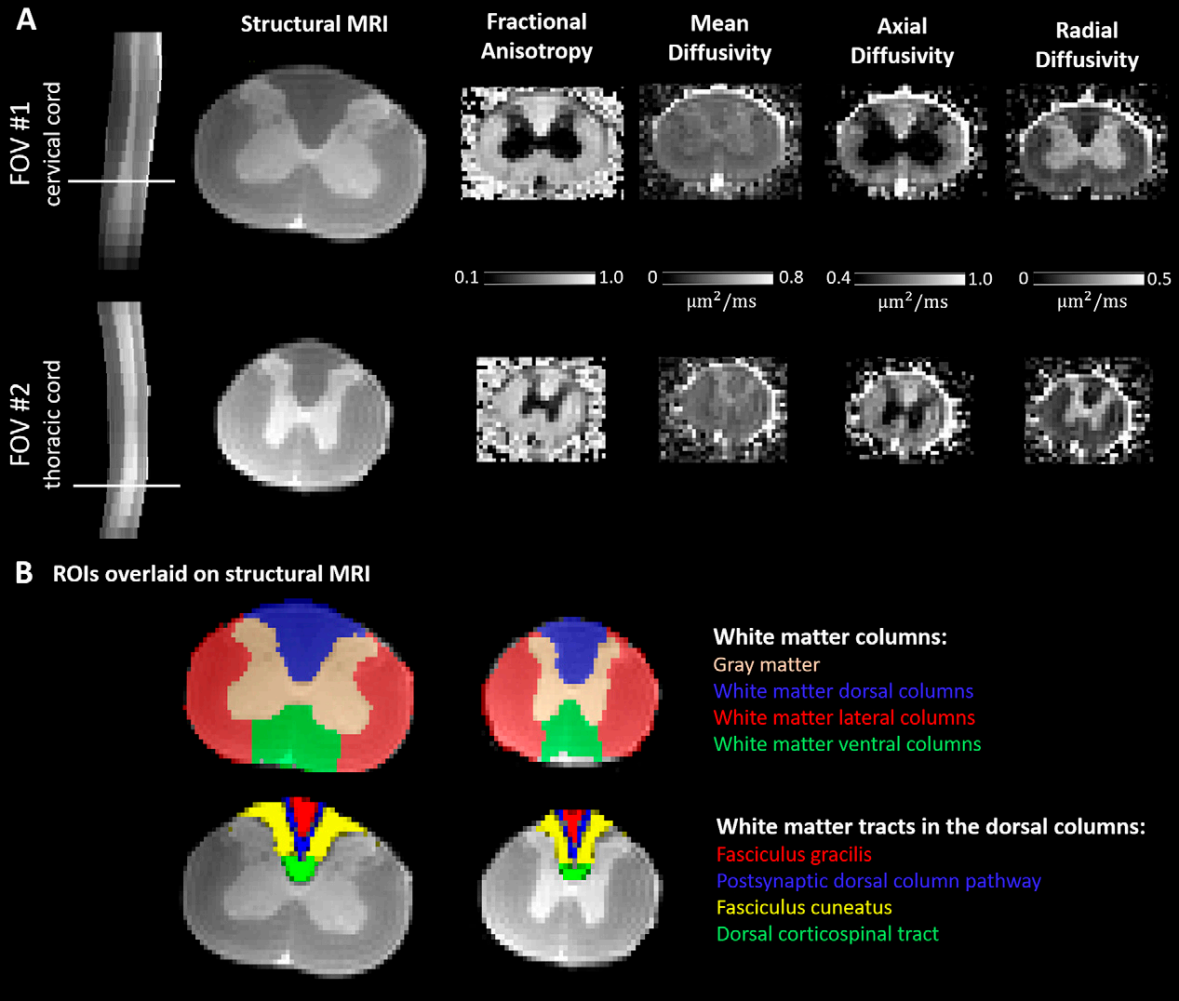
MRI of the spinal cord. **(A)** Each specimen underwent scanning in two sessions. The FOV of one session encompassed the more rostral part (roughly the cervical cord), while the FOV of the other session covered the more caudal part (roughly the upper and mid-thoracic cord) of the spinal cord. For both FOVs, shown are a sagittal slice acquired with the RARE-VTR sequence (TR = 5000 ms), as well as a representative coronal slice (location indicated by a white line on the sagittal image) of the same image. Additionally, coronal slices of DTI maps including the mean, axial, and radial diffusivities, and the fractional anisotrophy, are provided. All images are displayed in neurological format. **(B)** Location of the ROIs on representative cervical and thoracic coronal slices. The ROIs included the WM columns (dorsal, lateral, and ventral) and the gray matter, defined as binary masks, as well as the WM tracts within the dorsal columns (fasciculus gracilis, postsynaptic dorsal column pathway, fasciculus cuneatus, and dorsal corticospinal tract), defined as probabilistic maps (here thresholded at 0.5). The binary masks were eroded by one voxel when used to extract average values of DTI metrics. The T1-weighted image was used to obtain forward and backward warping fields between the native space and the rat spinal cord template. The backward warping field was used to warp the ROIs from the template to the native space. DTI, diffusion tensor imaging; FOV, field of view; MRI, magnetic resonance imaging; ROI, region of interest; WM, white matter.

**FIG. 3.**
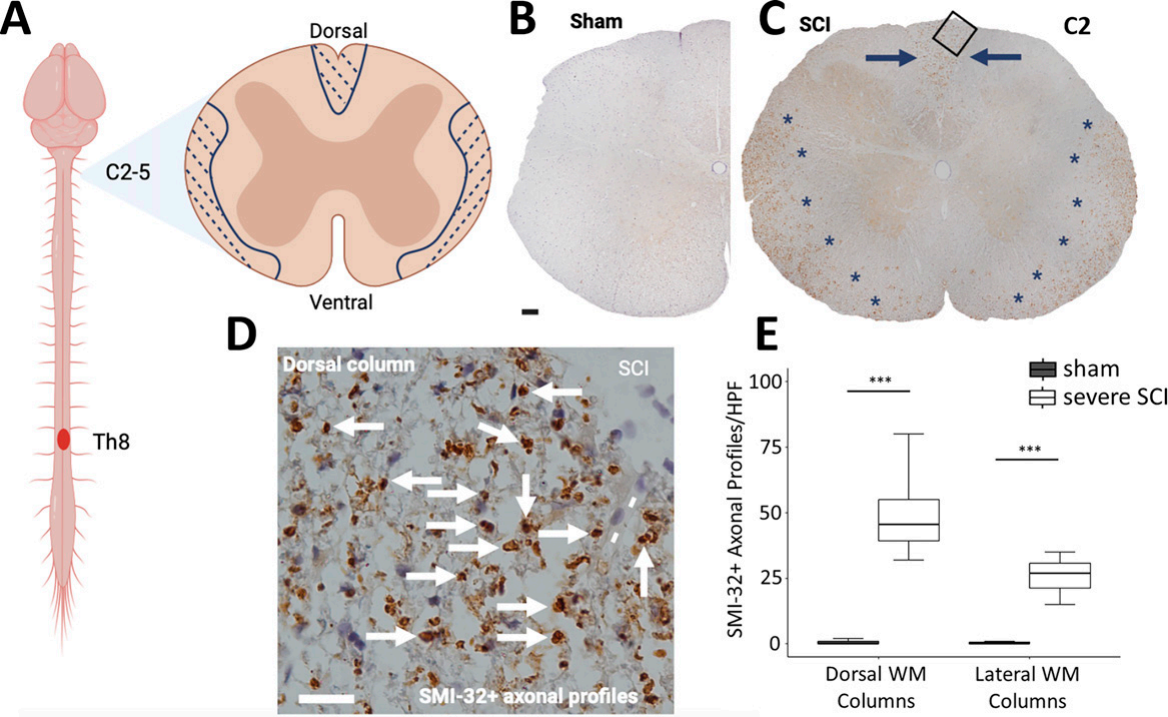
SMI-32 immunohistochemistry at 56 dpi. **(A)** Contusion SCI was induced at thoracic vertebral level T8 and tissue sections were extracted from above the lesion site, at C2–C5. **(B)** Representative image of SMI-32 labeling in a sham animal, showing only negligible SMI-32+ axonal profiles. **(C)** Representative image of SMI-32 labeling in a severe SCI animal, with SMI-32+ axonal profiles (dark brown) mainly localized in the dorsal WM (indicated by blue arrows) and in the medio- and ventrolateral regions (marked by blue stars). **(D)** Higher magnification illustrating SMI-32+ axonal profiles in the dorsal columns (white arrows) from a region depicted in **(C)** (black square). Note the proximity of degenerating SMI-32+ axonal profiles to pial vessels (white lines) acting as an anatomical interface to enable further vascular signaling and/or the shuttling of inflammatory mediators. **(E)** Quantification of SMI-32+ axonal profiles within the corresponding dorsal and the dorso-/mediolateral WM regions at 56 dpi, presented as box plots (SCI, *n =* 5; sham, *n =* 4). The numbers of SMI-32+ axonal profiles were counted within a single representative 5-μm section in five HPF (×400 with an eyepiece grid representing 0.0625 mm^2^) in both regions, and an average across HPFs was computed. *t*-Tests revealed a significant increase in SMI-32+ axonal profiles in both regions. Whiskers indicate 1.5 times the IQR or minimum/maximum values. Significance code: *p* < 0.001 (***). Bar in **(B)** represents 100 µm, and in **(D)** 20 µm. Part of **(A)** was created with BioRender.com. dpi, days post-injury; HPF, high-power field; IQR, interquartile range; SCI, spinal cord injury; WM, white matter.

#### Experimental design and statistical analyses

Statistical analyses were performed in Graph Pad Prism 9.2.0. After confirming normal distribution, two *t*-tests were performed to compare the number of SMI-32+ axonal profiles between the SCI and sham groups within the dorsal and lateral columns, respectively.

### Experiment 2: Investigating the effect of severity and time after injury on remote above-lesion axonal degeneration using SMI-32 immunohistochemistry

#### Contusion SCI rat model

The experiment involved 72 female Sprague Dawley (Charles River Laboratories, Wilmington, MA) adult rats weighing 200–250 g. Rats underwent dorsal laminectomy and graded contusion SCI at thoracic vertebral level T8 using the MASCIS impactor (W.M. Keck Center for Collaborative Neuroscience; Piscataway, NJ) as previously described.^15–17^ Animals were placed under 5% isoflurane and the spinal column was exposed using sterile procedures. A dorsal laminectomy was done with the dura remaining intact. A contusion injury was delivered by dropping a 10 g rod from a height of 12.5, 25.0, or 50.0 mm to induce mild (*n =* 18), moderate (*n =* 18), or severe injury (*n =* 18), respectively. Sham animals (*n =* 18) also underwent laminectomy with the weight briefly touched to the cord (instead of dropping). After the injury, the muscles were sutured in layers. Rats were placed on post-operative care, including twice-daily bladder expression and a once-daily dose of enrofloxacin (10 mg/kg subcutaneously; Bayer Healthcare LLC, Shawnee Mission, KS), buprenorphine hydrochloride (0.1–0.5 mg/kg subcutaneously; Rickitt Benckiser Health Care Ltd, Hull, U.K.), and 6 mL of lactated Ringer’s solution. Animals were kept under post-operative care until bladder function returned and no signs of infection or stress were evident. Using a predetermined schedule, rats for each injury severity were sacrificed at either 2, 30, or 90 dpi, resulting in *n =* 6 for each severity and time point. All animal procedures were approved by the Institutional Animal Care and Use Committees at the Medical College of Wisconsin and the Clement J. Zablocki Veterans’ Affairs Medical Center.

#### Behavioral testing

Rats were assessed for post-injury locomotor function according to the Basso, Beattie, and Bresnahan (BBB) score, where a score of 0 represents flaccid paralysis and a score of 21 represents normal locomotion.^18^ Open field locomotion was filmed and scored by two raters blinded to the data, which were averaged for final analysis. BBB scoring was performed at 1 and 7 dpi and weekly until 84 dpi.

#### SMI-32 immunohistochemistry

Animals were euthanized with 0.22 mL/kg sodium pentobarbital (Merck & Co., Inc., Madison, NJ) and perfused with phosphate buffer saline through the left cardiac ventricle followed by 10% formalin (Sigma-Aldrich, Co., St. Louis, MO). The C5 segment of the spinal cord was excised, immersed in formalin (Sigma-Aldrich, Co.) for 48 h, and embedded in paraffin. The tissue was sliced using a microtome in 5-μm sections, mounted on slides, deparaffinized, and rehydrated. Antigen retrieval was performed in a sodium citrate solution in a pressure cooker. Sections were blocked in 2% blocking buffer (Thermo Scientific, Rockford, IL) for 5 min at RT, and were incubated at RT overnight with the primary antibody against nonphosphorylated neurofilament (SMI-32; 1:500; Biolegend, San Diego, CA). After rinsing, goat anti-mouse secondary antibody conjugated with Alexa Fluor (1:500, Life Technologies, Eugene, OR) was applied. After washing, the sections were cover-slipped with Vectashield Mounting Medium with 4′,6′-diamidino-2-phenylindole (Vector Laboratories, Inc., Burlingame, CA).

**FIG. 4.**
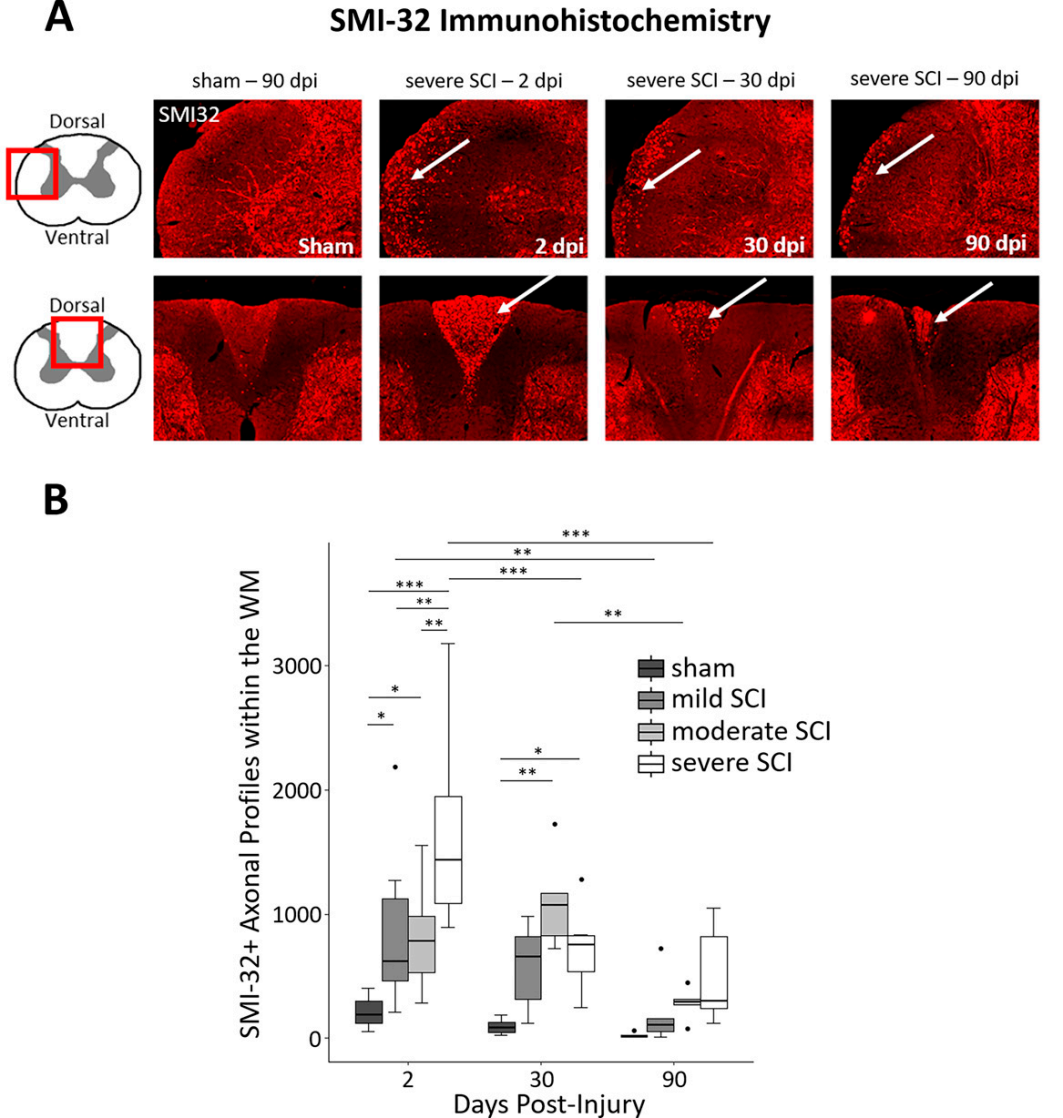
SMI-32 immunohistochemistry over time. **(A)** Representative images of SMI-32 labeling. Lateral WM is shown in the top row and dorsal WM in the bottom row comparing a sham specimen (left) with 2, 30, and 90 dpi severe animals (from left to right, respectively). In each animal, a single 5-μm section was obtained from spinal segment C5. The dorsal and lateral WM were the regions with the most intense SMI-32 labeling. At 90 dpi, labeling was constrained to a narrow rim along the edge of the spinal cord (upper right). **(B)** Box plot showing the total number of SMI-32-labeled axonal profiles within the entire WM of a single 5-μm section (i.e., no subsampling or extrapolation) across animal groups with varying severity (*n =* 6 for each group). There was an increase in labeling intensity with injury severity, as tested using two-way ANOVA, which was most evident at 2 dpi. This effect was smaller, yet still present at 90 dpi. The number of SMI-32-labeled axonal profiles diminished over time. Highlighted are the significant differences (*p* < 0.05) either between groups at the same time point or between time points within the same group. Whiskers indicate 1.5 times the IQR or minimum/maximum values. Dots represent outliers that fall below Q1—1.5 times the IQR or above Q3 + 1.5 times the IQR (where Q1 and Q3 are the first and third quartiles, respectively, and IQR = Q3—Q1). Significance codes: *p* < 0.05 (*), *p* < 0.01 (**), *p* < 0.001 (***). ANOVA, analysis of variance; IQR, interquartile range; SCI, spinal cord injury; WM, white matter.

A single 5-μm section was analyzed using a confocal microscope (Leica Microsystems, Inc., Model TCS SP8, Buffalo Grove, IL) at 20× magnification and a resolution of 0.57 μm per pixel (Fig. 4A). The entire section was imaged (i.e., no subsampling) and processed in ImageJ (Wayne Rasband, National Institutes of Health, Bethesda, MD), using the *analyze particles* plugin. Images were thresholded using the maximum entropy of the image histogram,^19^ which is a statistical measure of randomness. A region of interest (ROI) was manually drawn to segment the white matter (WM). Axonal damage was quantified as the total count of supra-threshold SMI-32+ axonal profiles within the WM.

#### Experimental design and statistical analyses

Statistical analyses were performed in R (v4.2.0). The number of SMI-32-labeled axonal profiles was compared using a two-way analysis of variance (ANOVA) with time since injury and injury severity as between-subject factors. The interaction between these two variables was also modeled to account for potentially varying group differences at different time points. *Post hoc* pairwise group comparisons were done using Fisher’s least significant difference method. BBB scores were modeled using repeated measures ANOVA with time since injury, injury severity, and their interaction as between-subject factors. Relationships between BBB scores and the number of SMI-32-labeled axonal profiles at 2, 30, and 90 dpi were assessed using Pearson’s product moment correlation.

### Experiment 3: Investigating the imaging correlates of remote above-lesion axonal degeneration using ex vivo MRI

#### Contusion SCI rat model

Twenty-seven female Sprague Dawley rats were included in the experiment, comprising mild SCI (*n =* 7), moderate SCI (*n =* 8), severe SCI (*n =* 5), and sham (*n =* 7). At 90 dpi, the rats were sacrificed, and their spinal cord was utilized for *ex vivo* MRI. The induction of contusion SCI, post-operative care, and animal sacrifice procedures were identical to those described in Experiment 2.

#### Image acquisition

*Ex vivo* MRI was performed on a 9.4T Bruker BioSpec 94/30 small animal MR scanner (Bruker BioSpin MRI, Ettlingen, Germany) equipped with a BGA-S gradient system, a linearly polarized volume resonator coil for homogeneous transmission, a 2 × 2 phased-array cryogenic surface coil for reception, and operated with Paravision 6.0.1 software. Spinal cord specimens were placed into tubes filled with Fomblin® to limit susceptibility artifacts. As the specimens were longer than the maximum field of view (FOV) of the coil, each specimen was scanned in two sessions with one session covering the upper and mid-thoracic and the other session the cervical spinal cord (Fig. 2A). Shimming was performed using the mapshim protocol in an ellipsoidal reference volume covering the spinal cord.

Structural images were acquired using the Rapid Acquisition with Relaxation Enhancement at Variable Repetition Time (RARE-VTR) sequence with the following sequence parameters: 25 coronal slices, slice thickness of 0.8 mm (no gap), in-plane FOV of 15.6 × 12 mm^2^, in-plane resolution of 78 × 94 μm^2^, repetition times (TR) of 450, 600, 800, 1500, 3000, and 5000 ms, echo time (TE) of 7 ms, bandwidth of 100 kHz, phase-encoding direction along A-P, and acquisition time of 24 min (see Fig. 2A for example images).

Diffusion-weighted images were acquired by employing Stejskal–Tanner diffusion gradients along 17 unique diffusion directions and at *b* values of 800 s/mm^2^ and 1600 s/mm^2^. Additional five nondiffusion-weighted images were acquired, making the diffusion MRI dataset consist of 39 images in total. The diffusion module was embedded in a 3D four-shot echo planar imaging sequence with the following sequence parameters: FOV of 7.5 × 30 × 7.5 mm^3^, isotropic resolution of 150 μm^3^, TR of 750 ms, TE of 20.25 ms, flip angle of 90°, bandwidth of 250 kHz, phase-encoding direction along A-P, and acquisition time of 1 h 38 min.

#### Image processing

##### Spatial processing

A binary spinal cord mask was obtained by segmenting the spin echo image with TR = 5000 ms for spinal cord using the *Propseg* algorithm.^20^ The image was spatially normalized to a rat spinal cord atlas of WM microstructure, constructed from high-resolution electron microscopy at each spinal level.^21^ Normalization was achieved in Spinal Cord Toolbox by nonlinearly registering (warping) the image to the map of myelin volume fraction, which involves manual placement of the spinal cord midpoint at each spinal level, automated cord straightening, rigid alignment, and nonlinear warping to the template.^22^ Both forward and backward warping fields were obtained.

##### Regions of interest

Binary masks of GM and WM were obtained from the rat spinal cord atlas, and WM was further subsegmented into dorsal, lateral, and ventral columns. The lateral columns were separated from both the ventral and dorsal columns by drawing a vertical line from the dorsal apex of the GM to the edge of the spinal cord on both sides (Fig. 2B).^21^ We also utilized the probabilistic masks of the four WM tracts that together constitute the dorsal column: the fasciculus gracilis, the postsynaptic dorsal column pathway, the fasciculus cuneatus, and the dorsal corticospinal tract (Fig. 2B).

##### Computation of cross-sectional areas

All binary masks were transformed into the native space by applying the backward warping field with nearest neighbor interpolation. Binary masks in the native space were used to compute cross-sectional areas of each ROI at each spinal level (C3–T6). As two images were acquired for each specimen, there was at least one overlapping spinal level at the cervical-thoracic junction. For the overlapping levels, the cross-sectional area was computed as the weighted average of the two cross-sectional area values, where the weights represented the number of overlapping slices in each image.

##### Tensor-based morphometry

Tensor-based morphometry was used to identify regional volumetric differences between groups. A voxel-wise Jacobian matrix was obtained from the spatial derivatives of the forward warping field, which contains information about the local stretching, shearing, and rotation. Then, the logarithm of the determinant of the Jacobian matrix (i.e., the logarithmic Jacobian determinant) was computed and subsequently smoothed using a Gaussian kernel with a full width at half maximum of 0.4 × 0.4 × 1 mm^3^. Higher values of the (logarithmic) Jacobian determinant indicate smaller local volumes, and vice versa.

##### Diffusion tensor imaging

The diffusion tensor model was fitted using a weighted least squares algorithm implemented in DIPY^23^ to derive maps of fractional anisotropy (FA), mean diffusivity (MD), axial diffusivity (AD), and radial diffusivity (RD) (Fig. 2A). Mean values were extracted from binary ROIs, while weighted averages were computed within probabilistic masks. Before metric extraction, the ROIs were transformed to native space using the backward warping field. To minimize partial volume effects, binary masks were eroded by one voxel. For the overlapping spinal levels, the image with superior quality was selected.

### Experimental design and statistical analyses

Statistical analyses were performed in R (v4.2.0). Linear mixed effect models, as implemented in the *nlme* (v3.1.157) library, were used for the analysis of *ex vivo* metrics (cross-sectional areas and DTI metrics). Fixed effects included the group (between-subject factor), spinal level (within-subject factor), and their interaction, while the specimens were treated as random effects. *Post hoc* tests, employing Bonferroni correction for multiple comparisons (*n =* 3), were conducted using the *emmeans* library (v.1.7.3) to assess group differences between mild SCI vs. sham, moderate SCI vs. sham, and severe SCI vs. sham. Levels were grouped into four segments to reduce the number of comparisons: C3–C5, C6–C8, T1–T3, and T4–T6. The level of significance was set to *p* < 0.05. Due to low quality, one sham and one severe SCI specimen were excluded from the analysis of cross-sectional areas, and two sham and two severe SCI specimens from the analysis of DTI metrics. The cervical segment was not available in an additional severe SCI specimen. Tensor-based morphometry was used to test for voxel-wise group differences in the logarithmic Jacobian determinants using ANOVA in SPM12. We computed maps of F-statistics to test for the overall group effect, and t-statistics for *post hoc* pairwise group differences. Maps of F- and t-statistics were thresholded at *p* < 0.001 (uncorrected), followed by a peak-level threshold of *p* < 0.05 (family-wise error [FWE] corrected) based on Gaussian Random Field theory. Relationships between the BBB scores and the *ex vivo* MRI metrics were assessed using Pearson’s product moment correlation.

## Results

### Spatial distribution of remote above-lesion axonal degeneration (Experiment 1)

In sham animals, SMI-32 is predominantly expressed by larger GM neurons, while WM axons exhibit minimal presence of SMI-32+ axonal profiles (Fig. 3B). Following SCI, SMI-32 expression emerges *de novo* in WM tracts, presenting a characteristic pattern far above the lesion site (C2–C5). The most pronounced accumulation of SMI-32+ axonal profiles was observed within the fasciculus gracilis and the periphery of the medio- and ventrolateral columns (Fig. 3C). *t*-Tests revealed a higher number of SMI-32+ axonal profiles per HPF in the severe SCI group within the fasciculus gracilis in the dorsal WM (47.8 ± 11.8, vs. sham: *p* < 0.001) and the dorso-/mediolateral parts of the lateral WM columns (26.2 ± 5.5, vs. sham: *p* < 0.001) (Fig. 3E).

### Effect of severity and time after injury on remote above-lesion axonal degeneration (Experiment 2)

At 2 dpi, intense SMI-32 labeling appeared most prominently in the fasciculus gracilis of the dorsal column and on the periphery of the medio- and ventrolateral columns (Fig. 4). At 90 dpi, SMI-32 labeling was limited to a narrow rim on the edge of the cord in the ventrolateral region and atrophy was prominent in the fasciculus gracilis compared with earlier time points.

Two-way ANOVA revealed a significant effect of injury severity (*F*[3,62] = 10.07, *p* < 0.001) and time after injury (*F*[2,63] = 12.91, *p* < 0.001) on SMI-32 labeling (Fig. 4B). The interaction between time and injury severity was also significant (*F*[6,59] = 2.52, *p* = 0.032). At 2 dpi, all SCI groups had more SMI-32-labeled axonal profiles than the sham group (severe vs. sham: *p* < 0.001; moderate vs. sham: *p* = 0.021; mild vs sham: *p* = 0.010). In addition, the severe SCI group had more SMI-32-labeled axonal profiles than the mild (*p* = 0.003) and the moderate SCI group (*p* = 0.001). At 30 dpi, only the severe (*p* = 0.031) and moderate SCI groups (*p* = 0.001) showed more SMI-32 labeling than shams. No pairwise differences were observed at 90 dpi. When investigating the effect of time after injury in each group separately, the number of SMI-32-labeled axonal profiles decreased between 2 and 30 dpi in the severe SCI group (*p* < 0.001), between 30 and 90 dpi in the moderate SCI group (*p* = 0.005), and between 2 and 90 dpi in the mild (*p* = 0.008) and the severe SCI group (*p* < 0.001).

### Cross-sectional tissue area and volumetric assessments of remote above-lesion axonal degeneration (Experiment 3)

**FIG. 5.**
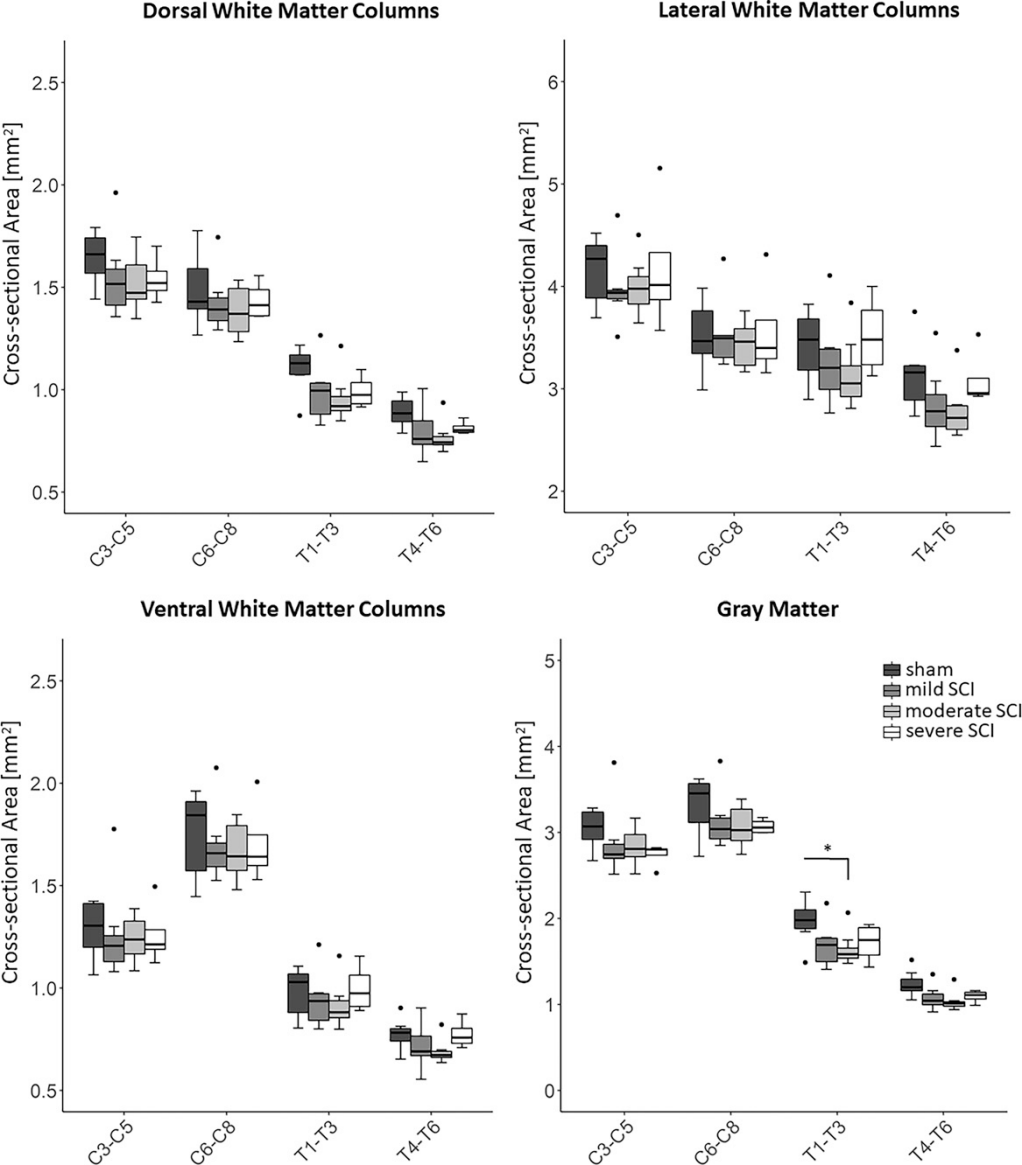
Cross-sectional area of spinal cord regions. Box plots comparing the cross-sectional areas of four ROIs (dorsal, lateral, and ventral white matter columns as well as gray matter) between groups of varying severity (sham, *n =* 7; mild SCI, *n =* 7; moderate SCI, *n =* 8; severe SCI, *n =* 5), at rostral spinal cord segments C3-T6. The location of the ROIs is shown in Fig. 2B. Between-group comparisons were done using linear mixed effects models followed by *post hoc* tests. Whiskers indicate 1.5 times the IQR or minimum/maximum values. Dots represent outliers that fall below Q1—1.5 times the IQR or above Q3 + 1.5 times the IQR (where Q1 and Q3 are the first and third quartiles, respectively, and IQR = Q3—Q1). Significance code: *p* < 0.05 (*). IQR, interquartile range; ROI, region of interest; SCI, spinal cord injury.

**FIG. 6.**
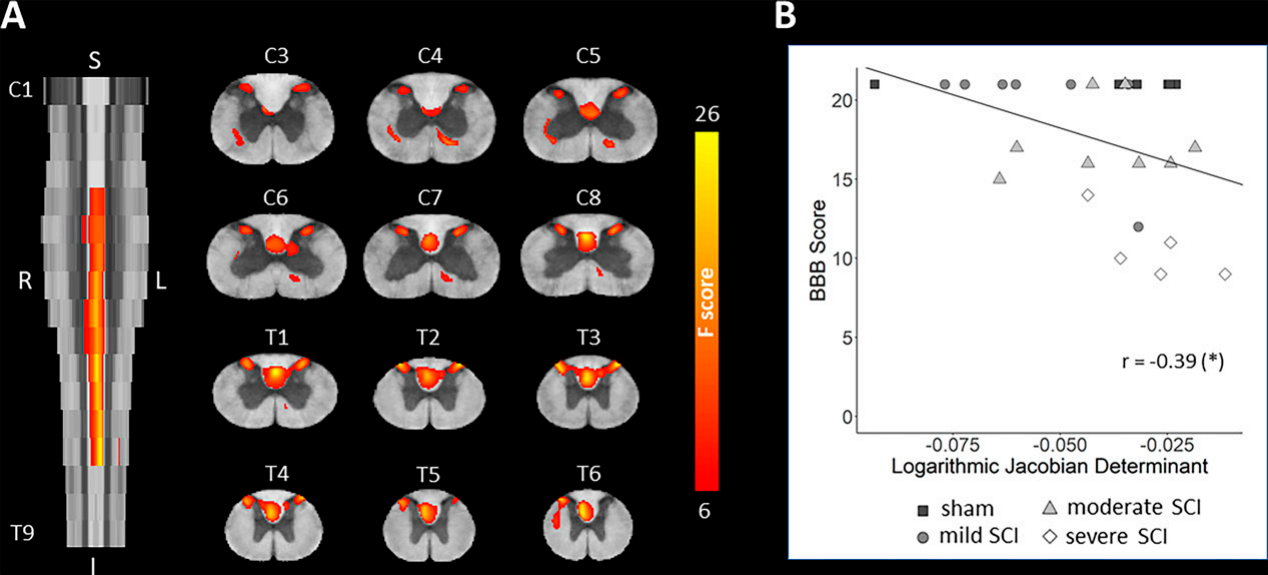
Tensor-based morphometry. **(A)** Group differences in the (logarithmic) Jacobian determinants were tested using ANOVA. *F-*scores, displayed as heatmap and thresholded at a cluster level *p* < 0.05 (family-wise error corrected), show voxels with significant group effect on the Jacobian determinants, which can be interpreted as severity-dependent local volume differences. The *F-*scores are overlaid on the rat spinal cord template and are shown both in the axial and coronal plane. **(B)** Scatter plot showing the negative correlation between the BBB score and the mean Jacobian determinant within the significant cluster in the dorsal columns, indicating smaller local volumes in animals with lower BBB scores. Significance code: *p* < 0.05 (*). ANOVA, analysis of variance; BBB, Basso, Beattie, and Bresnahan score.

The cross-sectional areas were significantly different across spinal levels, but not across groups. *Post hoc* analyses revealed that, compared with the sham group, the moderate SCI group had lower cross-sectional area of the GM at T1–T3 (−16.3%, *p* = 0.039) (Fig. 5, Table 1). Tensor-based morphometry demonstrated a significant group effect within three clusters. The first cluster is centered in the anterior aspect of the dorsal columns, largely overlapping with the dorsal corticospinal tract, but extending into the left and right dorsal GM horns as well (peak *F* value = 26.08, cluster size = 2231, *p* [FWE-corrected at cluster level] < 0.001), the second one at the anterior edge of the left GM horn (*F* [peak] = 16.55, cluster size = 131, *p* [FWE] = 0.005) and the third one at the anterior edge of the right GM horn (*F* = 12.89, cluster size = 111, *p* [FWE] = 0.010) (Fig. 6). *Post hoc* pairwise group comparisons did not reveal significant differences in the Jacobian determinants.

**Table 1.** Cross-Sectional Areas in White Matter Columns and Gray Matter (Experiment 3)

	ROI	Sham	Mild SCI	Moderate SCI	Severe SCI	Mild SCI-sham		Moderate SCI-sham		Severe SCI-sham	
						Diff.	*p*	Diff.	*p*	Diff.	*p*
	C3–C5										
	WM dor	1.64 ± 0.13	1.55 ± 0.21	1.52 ± 0.14	1.54 ± 0.12	−5.9%	0.314	−7.8%	0.157	−6.2%	0.535
Cross-sectional areas (mm^2^)	WM lat	4.15 ± 0.32	3.97 ± 0.36	3.99 ± 0.28	4.19 ± 0.68	−4.2%	1.000	−4.0%	1.000	0.9%	1.000
	WM ven	1.29 ± 0.14	1.26 ± 0.24	1.24 ± 0.11	1.26 ± 0.16	−2.1%	1.000	−3.5%	0.858	−2.1%	1.000
	GM	3.05 ± 0.24	2.88 ± 0.43	2.85 ± 0.22	2.74 ± 0.14	−5.4%	0.229	−6.6%	0.148	−10.1%	0.105
	C6–C8										
	WM dor	1.49 ± 0.17	1.43 ± 0.15	1.38 ± 0.12	1.44 ± 0.10	−4.3%	0.969	−7.4%	0.248	−3.8%	0.908
	WM lat	3.52 ± 0.35	3.52 ± 0.35	3.44 ± 0.22	3.57 ± 0.51	0.0%	1.000	−2.3%	1.000	1.3%	1.000
	WM ven	1.75 ± 0.21	1.69 ± 0.18	1.66 ± 0.14	1.70 ± 0.21	−3.0%	1.000	−4.8%	0.667	−2.3%	1.000
	GM	3.31 ± 0.34	3.13 ± 0.33	3.06 ± 0.25	3.07 ± 0.09	−5.5%	0.414	−7.5%	0.116	−7.2%	0.295
	T1–T3										
	WM dor	1.10 ± 0.11	0.99 ± 0.15	0.95 ± 0.11	0.99 ± 0.08	−10.3%	0.359	−13.3%	0.084	−10.0%	0.452
	WM lat	3.42 ± 0.34	3.26 ± 0.44	3.15 ± 0.34	3.52 ± 0.40	−4.6%	1.000	−7.9%	0.481	3.0%	1.000
	WM ven	0.98 ± 0.12	0.94 ± 0.14	0.91 ± 0.11	1.00 ± 0.12	−3.8%	1.000	−6.6%	1.000	2.2%	1.000
	GM	1.96 ± 0.26	1.69 ± 0.26	1.64 ± 0.19	1.72 ± 0.23	−14.1%	0.150	−16.3%	**0.039**	−12.5%	0.262
	T4–T6										
	WM dor	0.89 ± 0.07	0.80 ± 0.12	0.77 ± 0.08	0.81 ± 0.03	−10.6%	0.437	−13.7%	0.141	−8.7%	0.910
	WM lat	3.12 ± 0.34	2.84 ± 0.37	2.79 ± 0.28	3.09 ± 0.29	−9.0%	0.418	−10.8%	0.158	−1.0%	1.000
	WM ven	0.77 ± 0.08	0.72 ± 0.11	0.69 ± 0.06	0.77 ± 0.07	−7.5%	1.000	−10.9%	0.624	0.0%	1.000
	GM	1.24 ± 0.16	1.08 ± 0.14	1.04 ± 0.12	1.09 ± 0.08	−13.1%	0.488	−16.3%	0.226	−11.9%	0.888

Values represent mean and standard deviation across samples, separately for each level (C3–C5, C6–C8, T1–T3, T4–T6), ROI, and group (sham, *n =* 7; mild SCI, *n =* 7; moderate SCI, *n =* 8; severe SCI, *n =* 5). Significant *p* values (<0.05) are highlighted in bold.

GM, gray matter; ROI, region of interest; SCI, spinal cord injury; WM dor, dorsal white matter columns; WM lat, lateral white matter columns; WM ven, ventral white matter columns.

### Diffusion tensor imaging (Experiment 3)

**FIG. 7.**
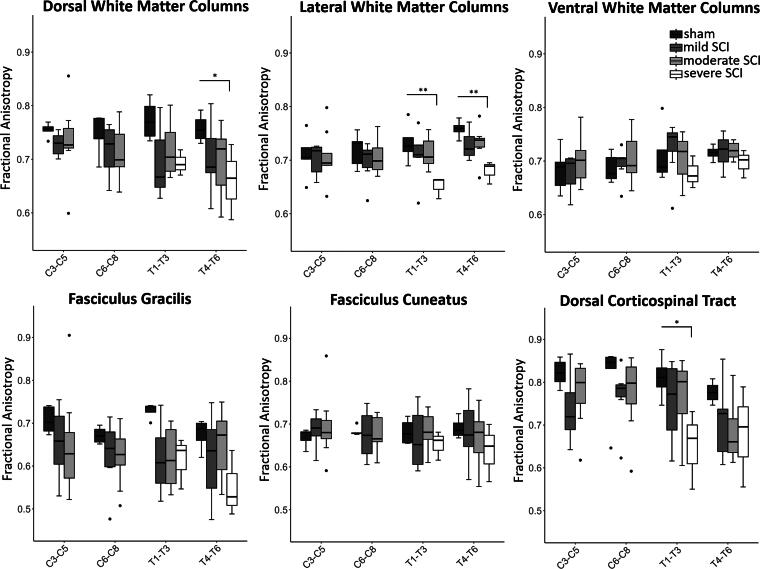
Fractional anisotropy in the white matter columns (dorsal, lateral, and ventral) and in specific tracts within the dorsal columns. Box plots comparing the fractional anisotropy between the sham and SCI groups of varying severity (sham, *n* = 7; mild SCI, *n* = 7; moderate SCI, *n* = 8; severe SCI, *n* = 5), at rostral spinal cord segments C3-T6, averaged within the ROIs. Due to dropouts, the sample size for the severe SCI group was smaller than three at C3–C5 and C6–C8, and hence were not included in the plot. The location of the ROIs is shown in Fig. 2B. Between-group comparisons were done using linear mixed effects models followed by *post hoc* tests. Whiskers indicate 1.5 times the IQR or minimum/maximum values. Dots represent outliers that fall below Q1—1.5 times the IQR or above Q3 + 1.5 times the IQR (where Q1 and Q3 are the first and third quartiles, respectively, and IQR = Q3—Q1). Significance codes: *p* < 0.05 (*), *p* < 0.01 (**). IQR, interquartile range; ROI, region of interest; SCI, spinal cord injury.

FA in the lateral columns (*p* = 0.019) and in the postsynaptic dorsal column pathway (*p* = 0.048) showed significant differences across groups. *Post hoc* analysis revealed that, compared with the sham group, the severe SCI group exhibited lower FA in the dorsal columns at T4–T6 (−12.9%, *p* = 0.036) and in the lateral columns at T4–T6 (−10.3%, *p* = 0.008) and T1–T3 (−10.9%, *p* = 0.007) (Fig. 7, Table 2). Within the dorsal columns, the severe SCI group also showed lower FA in the postsynaptic dorsal column pathway at T4–T6 (−13.6%, *p* = 0.037) and in the dorsal corticospinal tract at T1–T3 (−19.8%, *p* = 0.045). Furthermore, the severe SCI group showed lower AD in the dorsal columns at T4–T6 (vs. sham, −22.4%, *p* = 0.049), as well as in the postsynaptic dorsal column pathway at T4–T6 (−24.5%, *p* = 0.031). No significant differences in MD and RD were observed between the sham group and any of the SCI groups.

**Table 2.** Fractional Anisotropy Values Measured in White Matter Columns, Specific Tracts within the Dorsal Columns, and Gray Matter (Experiment 3)

	ROI	Sham	Mild SCI	Moderate SCI	Severe SCI	Mild SCI-sham		Moderate SCI-sham		Severe SCI-sham	
						Diff.	*p*	Diff.	*p*	Diff.	*p*
	C3–C5										
Fractional anisotropy	WM dor	0.75 ± 0.01	0.73 ± 0.02	0.73 ± 0.07	—	−3.5%	0.808	−2.7%	1.000	—	—
	WM lat	0.71 ± 0.04	0.70 ± 0.03	0.71 ± 0.05	—	−1.0%	1.000	−0.3%	1.000	—	—
	WM ven	0.68 ± 0.04	0.68 ± 0.04	0.70 ± 0.04	—	−0.8%	1.000	2.9%	0.960	—	—
	WM FG	0.71 ± 0.03	0.65 ± 0.09	0.65 ± 0.12	—	−7.5%	0.464	−8.1%	0.201	—	—
	WM PSdC	0.78 ± 0.02	0.75 ± 0.04	0.75 ± 0.08	—	−4.0%	0.786	−3.4%	0.752	—	—
	WM FC	0.67 ± 0.02	0.69 ± 0.04	0.69 ± 0.08	—	2.5%	1.000	3.9%	1.000	—	—
	WM dCST	0.82 ± 0.03	0.74 ± 0.08	0.78 ± 0.08	—	−10.3%	0.289	−5.6%	1.000	—	—
	GM	0.33 ± 0.08	0.32 ± 0.03	0.37 ± 0.13	—	−2.6%	1.000	13.4%	1.000	—	—
	C6–C8										
	WM dor	0.74 ± 0.04	0.72 ± 0.05	0.71 ± 0.05	—	−3.6%	0.679	−4.1%	0.732	—	—
	WM lat	0.72 ± 0.03	0.70 ± 0.04	0.71 ± 0.03	—	−3.2%	0.307	−1.8%	1.000	—	—
	WM ven	0.69 ± 0.03	0.69 ± 0.03	0.70 ± 0.04	—	1.1%	1.000	2.5%	1.000	—	—
	WM FG	0.67 ± 0.02	0.62 ± 0.09	0.62 ± 0.07	—	−7.0%	0.563	−7.1%	0.732	—	—
	WM PSdC	0.75 ± 0.02	0.71 ± 0.07	0.71 ± 0.05	—	−5.8%	0.303	−6.2%	0.325	—	—
	WM FC	0.68 ± 0.01	0.68 ± 0.06	0.68 ± 0.04	—	−1.1%	1.000	−0.9%	1.000	—	—
	WM dCST	0.81 ± 0.09	0.77 ± 0.08	0.77 ± 0.09	—	−5.1%	1.000	−4.3%	1.000	—	—
	GM	0.34 ± 0.06	0.33 ± 0.03	0.34 ± 0.05	—	−1.9%	1.000	2.4%	1.000	—	—
	T1–T3										
	WM dor	0.77 ± 0.04	0.69 ± 0.07	0.72 ± 0.05	0.69 ± 0.02	−10.2%	0.051	−7.2%	0.231	−10.4%	0.108
	WM lat	0.73 ± 0.04	0.71 ± 0.05	0.71 ± 0.03	0.65 ± 0.02	−3.0%	0.905	−2.5%	1.000	−10.9%	**0.007**
	WM ven	0.71 ± 0.06	0.72 ± 0.05	0.71 ± 0.04	0.68 ± 0.03	1.7%	1.000	−0.7%	1.000	−4.8%	0.565
	WM FG	0.73 ± 0.02	0.62 ± 0.08	0.62 ± 0.07	0.61 ± 0.06	−15.3%	0.071	−15.3%	0.063	−15.7%	0.149
	WM PSdC	0.79 ± 0.02	0.69 ± 0.07	0.72 ± 0.05	0.71 ± 0.03	−12.2%	**0.026**	−9.0%	0.120	−9.9%	0.181
	WM FC	0.68 ± 0.03	0.67 ± 0.07	0.68 ± 0.04	0.65 ± 0.03	−2.4%	1.000	0.4%	1.000	−4.3%	1.000
	WM dCST	0.81 ± 0.05	0.75 ± 0.10	0.77 ± 0.08	0.65 ± 0.09	−7.1%	1.000	−5.3%	1.000	−19.8%	**0.045**
	GM	0.40 ± 0.08	0.45 ± 0.07	0.43 ± 0.09	0.40 ± 0.10	11.9%	1.000	7.3%	1.000	0.0%	1.000
	T4–T6										
	WM dor	0.76 ± 0.03	0.70 ± 0.07	0.69 ± 0.07	0.66 ± 0.07	−7.2%	0.285	−8.3%	0.261	−12.9%	**0.036**
	WM lat	0.76 ± 0.02	0.73 ± 0.03	0.73 ± 0.03	0.68 ± 0.02	−4.0%	0.542	−3.6%	0.650	−10.3%	**0.008**
	WM ven	0.72 ± 0.01	0.72 ± 0.03	0.72 ± 0.02	0.70 ± 0.03	0.3%	1.000	0.6%	1.000	−2.6%	1.000
	WM FG	0.67 ± 0.04	0.62 ± 0.10	0.65 ± 0.08	0.55 ± 0.08	−8.3%	0.767	−3.6%	1.000	−18.2%	0.107
	WM PSdC	0.78 ± 0.02	0.70 ± 0.07	0.72 ± 0.07	0.67 ± 0.07	−9.8%	0.089	−8.0%	0.337	−13.6%	**0.037**
	WM FC	0.69 ± 0.03	0.68 ± 0.07	0.67 ± 0.07	0.64 ± 0.07	−1.0%	1.000	−3.5%	1.000	−7.7%	0.385
	WM dCST	0.78 ± 0.03	0.71 ± 0.09	0.64 ± 0.15	0.68 ± 0.12	−9.1%	0.431	−16.9%	0.086	−12.4%	0.647
	GM	0.47 ± 0.09	0.52 ± 0.06	0.51 ± 0.09	0.45 ± 0.06	10.3%	0.934	7.7%	0.894	−4.7%	1.000

Values represent mean and standard deviation across samples, separately for each level (C3–C5, C6–C8, T1–T3, T4–T6), ROI, and group (sham, *n =* 7; mild SCI, *n =* 7; moderate SCI, *n =* 8; severe SCI, *n =* 5). Significant *p* values (<0.05) are highlighted in bold. No descriptive or inferential statistics were done for groups with a sample size smaller than three (affecting the severe SCI group at C3–C5 and C6–C8 for the analysis of fractional anisotropy).

GM, gray matter; ROI, region of interest; SCI, spinal cord injury; WM dor, dorsal white matter columns; WM dCST, dorsal corticospinal tract; WM FC, fasciculus cuneatus; WM FG, fasciculus gracilis; WM lat, lateral white matter columns; WM PSdC, postsynaptic dorsal column pathway, WM ven, ventral white matter columns.

### Locomotor function and its relationship to axonal markers and ex vivo MRI metrics (Experiments 2 and 3)

**FIG. 8.**
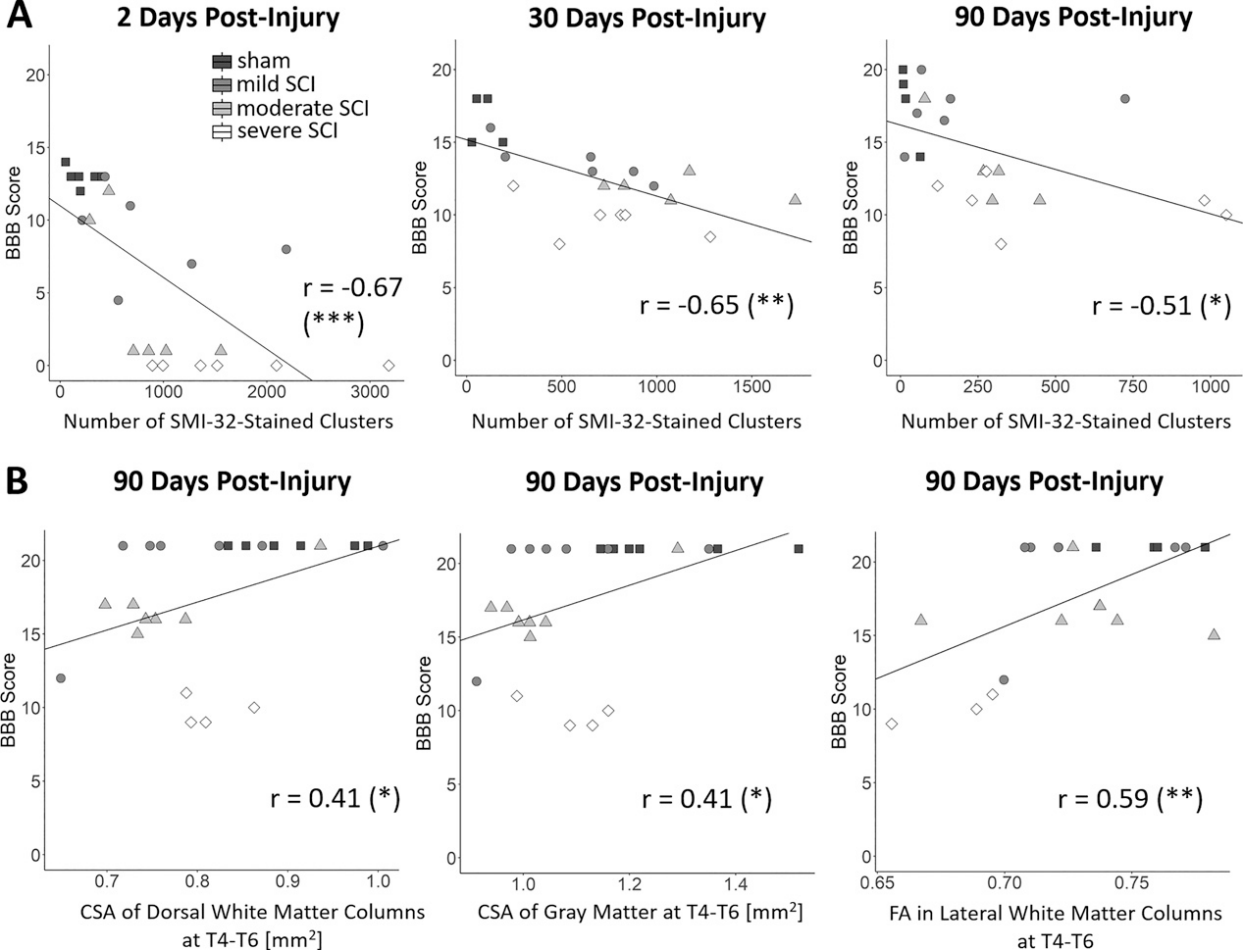
Associations between locomotion, SMI-32 staining, and MRI metrics. **(A)** Scatter plots depicting the negative correlations (Pearson correlation coefficient) between the BBB score and the number of SMI-32-labeled axonal profiles within the entire WM at 2, 30, and 90 dpi (*n =* 6 for each severity). **(B)** Scatter plots depicting the positive correlations between the BBB score and *ex vivo* MRI metrics at T4–T6 including the CSA of the dorsal white matter columns and the gray matter, and the fractional anisotropy within the lateral white matter columns (sham, *n =* 7; mild SCI, *n =* 7; moderate SCI, *n =* 8; severe SCI, *n =* 5). Significance codes: *p* < 0.05 (*), *p* < 0.01 (**), *p* < 0.001 (***). dpi, days post-injury; CSA, cross-sectional area; MRI, magnetic resonance imaging; BBB, Basso, Beattie, and Bresnahan.

BBB scores showed a negative correlation with the number of SMI-32-labeled axonal profiles within the entire WM at C5 at 2 dpi (*r* = −0.67, *p* < 0.001), 30 dpi (*r* = −0.65, *p* = 0.002), and 90 dpi (*r* = −0.51, *p* = 0.018) (Fig. 8A). When relating to *ex vivo* MRI metrics, BBB scores correlated positively with the cross-sectional area of the dorsal columns at T4–T6 (*r* = 0.41, *p* = 0.044) and T1–T3 (*r* = 0.40, *p* = 0.049), the cross-sectional area of the GM at T4–T6 (*r* = 0.41, *p* = 0.049), the FA within the lateral columns at T4–T6 (*r* = 0.59, *p* = 0.005), and the AD within the dorsal columns at T4–T6 (*r* = 0.55, *p* = 0.009), C6–C8 (*r* = 0.54, *p* = 0.011), and C3–C5 (*r* = 0.54, *p* = 0.013) (Fig. 8B). In the tensor-based morphometry analysis (Fig. 6), BBB scores correlated negatively with the logarithmic Jacobian determinants within the cluster covering the anterior aspect of the dorsal columns, extending into the left and right dorsal GM horns (*r* = −0.39, *p* = 0.047) (Fig. 6B). This indicates that lower BBB scores are associated with smaller local tissue volumes at these locations.

## Discussion

We demonstrated in a rat thoracic contusion SCI model that distal WM axons in the upper cervical cord (C2–C5) exhibit signs of degeneration following contusion SCI at the T8 vertebral level. Axonal degeneration was observed primarily within the fasciculus gracilis of the dorsal columns and the periphery of the medio- and ventrolateral columns (Figs. 3C and 4A). Importantly, key findings from our study (exploratory research) were reproduced in two separate experiments (Experiments 1 and 2) conducted at different centers by independent investigators (confirmatory research), which provides further validation and is considered crucial for establishing translational evidence.^24,25^ Despite the use of different impactors in the two experiments, a prior comparative study demonstrated that a contusion injury with a 250 kdyne impact force (Experiment 1) produces similar severe outcomes—in terms of cavity volume and locomotor function assessed by BBB scores and the ladder rung walking test—to those induced by dropping a 10 g rod from a height of 50.0 mm (severe SCI group in Experiment 2).^26^ Progressive axonal degeneration diminished over time but persisted as late as 90 dpi (Fig. 4). Rats with more severe injuries exhibited greater axonal degeneration (Fig. 4B). In line with the histological findings, diffusion MRI revealed lower fractional anisotropy within the dorsal and lateral columns in severe SCI (Fig. 7), while structural MRI demonstrated smaller area of GM in moderate SCI rats (Fig. 5). These observations closely mirror earlier MRI findings in individuals with SCI.^27^

### Dynamic remote axonal degeneration rostral to injury: insights from histology

We investigated disturbed axonal structure using SMI-32 immunohistochemistry, where the antibodies SMI-32 react with nonphosphorylated epitopes of neurofilament.^28^ Under physiological conditions, nonphosphorylated neurofilaments (SMI-32) are primarily detected in the cell body and dendrites^29^; neurofilaments in healthy myelinated axons are heavily phosphorylated and not labeled by SMI-32 antibodies.^28–30^ However, in the case of axonal damage, SMI-32 is also detected within the axons.^29,31,32^ The sensitivity and specificity of SMI-32 antibodies for injured axons make it an ideal marker for identifying early stages of axonal damage, and it has been widely used in rat SCI models^4,33–35^ and human tissues.^3,29,31,32^

We found presence of nonphosphorylated neurofilaments within the axons at C5 as early as 2 days after a contusion injury at the T8 vertebral level (Fig. 4). This is consistent with previous findings in rats that underwent L_4_L_5_L_6_ dorsal radiculotomy, where anterograde degeneration was observed using SMI-32 staining near the injury site within hours, which progressed from the lower lumbar to the cervical levels in the dorsal columns within 25–28 h after injury, corresponding to a rate of advance of 2.5–3 mm/h.^4^ In the same rat SCI model, the initial breakdown of axoplasm in the central nervous system was found to be complete by 3 dpi.^33^ In line with this, a study on dorsal root axotomy in rats reported an accumulation of SMI-32 in the dorsal columns from 38 h to 3 dpi.^34^

We observed above-lesion axonal degeneration most prominently within the fasciculus gracilis of the dorsal columns (Figs. 3C and 4A). This finding is not surprising, as the contusion directly impacted the fasciculus gracilis, and aligns with previous studies on rat contusion SCI models.^4,33,34,36,37^ In contrast, the fasciculus cuneatus, located more laterally and containing afferents from the upper trunk and extremities (C1 to approximately T6), appeared largely unaffected in the SMI-32 histological sections. We also detected axonal degeneration in the medio- and ventrolateral columns, which contain both ascending sensory (spinothalamic and spinocerebellar tracts) and descending motor tracts (raphespinal and reticulospinal tracts). We argue that the detected axonal degeneration within the fasciculus gracilis and the medio- and ventrolateral columns indicates anterograde degeneration of ascending sensory fibers, although other mechanisms might also occur. Degeneration within the lateral columns might also be attributed to retrograde degeneration of descending motor tracts, a process where the proximal segment of the severed axons gradually retracts toward the cell body (axonal dieback).^38^ However, we consider this scenario unlikely as axonal dieback has been observed only within a few millimeters from the lesion in the corticospinal tract of rat SCI models.^5,39,40^

Following the rapid axonal disintegration, characterized by the large number of SMI-32+ axonal profiles at 2 dpi, we observed reduced but persistent SMI-32 staining at 30 dpi, which remained detectable even at 90 dpi (Fig. 4). The prolonged presence of neurofilament epitopes has been previously noted in degenerating tracts,^4,41,42^ reflecting the slow debris clearance mechanisms commonly observed in the central nervous system.^33,43,44^ In close alignment with our findings, studies investigating dorsal column degeneration following dorsal radiculotomy or dorsal root axotomy in rats also reported, after the initial accumulation of SMI-32 staining in the first few days following injury, reduced but persistent SMI-32+ debris at 30 dpi^34^ and 90 dpi.^4,33^ During the same timeframe, all SCI animals in our cohorts, regardless of severity, demonstrated functional recovery. While recovery of function relies on axonal and synaptic plasticity, the intricate interplay between degenerative and regenerative mechanisms is still not fully understood.

### Remote WM degeneration rostral to injury: insights from imaging

Consistent with our histological findings, fractional anisotropy (FA) was lower in the dorsal and lateral columns rostral (T1–T6 spinal levels) to the injury site (appr. T10 spinal level) in severe SCI rats, compared with sham animals (Fig. 7 and Table 2). Within the dorsal columns, although not always statistically significant, the largest percentage differences in FA at T1–T6 were observed in the fasciculus gracilis (severe SCI vs sham; −15.7% to −18.2%) and the dorsal corticospinal tract (−12.4% to −19.8%), followed by the postsynaptic dorsal column pathway (−9.9% to −13.6%) and the fasciculus cuneatus (−4.3% to −7.7%). Signs of atrophy were also detected in the dorsal columns using tensor-based morphometry (Fig. 6A), with the cluster located mostly in the dorsal corticospinal tract, despite the cross-sectional area of the entire dorsal column not showing a significant difference between SCI and sham animals (Fig. 5 and Table 1). The fact that severe SCI rats had lower FA, but not cross-sectional area in the lateral columns indicates that DTI is more sensitive to neurodegenerative changes. While we did not find significant alterations in the ventral columns, we note that an *in vivo* imaging study conducted in a mouse contusion SCI model found significant FA decrease in the ventral columns at and near the injury site, albeit less prominent than in the dorsal columns.^45^

While the FA changes in the fasciculus gracilis are consistent with the contusion injury model and our histological SMI-32+ findings—showing degeneration and atrophy in the fasciculus gracilis—our MRI findings in the dorsal corticospinal tract are more difficult to interpret. We suspect that this discrepancy is at least partly due to the reliance of both atlas-based DTI analysis and tensor-based morphometry on accurate spatial normalization. Although our normalization procedure aligned individual spinal cords to the rat spinal cord atlas, it was not specifically optimized for aligning distinct WM tracts such as the fasciculus gracilis. This limitation is particularly relevant in the SCI animals, where the fasciculus gracilis is visibly atrophied and occupies significantly less volume at our endpoint (see white arrow in the microscopic image in Fig. 4A, severe SCI—90 dpi). As a result, some MRI findings may have been mislocalized to adjacent tracts. Additionally, the altered size and arrangement of WM tracts may have led to systematic differences in partial volume effects with gray matter—especially in the dorsal corticospinal tract, which are surrounded by gray matter on three sides.

Dorsal column atrophy is likely caused by axonal and myelin breakdown and phagocytosis of cellular debris by invading immune cells.^46^ Microglia and macrophages invade the dorsal columns after 4 weeks of survival and typically remain activated for a prolonged period of time.^46^ An increase in density of macrophages and microglia has been observed as far as 10.4 mm away from the injury epicenter in the rat spinal cord.^47,48^ Due to the progressive nature of phagocytosis, atrophy is most prominent in the chronic stage of injury.^49,50^ Tissue loss can somewhat be counteracted by proliferating astrocytes that fill the space left behind by removed axons.^46^

The percentage differences in FA within the dorsal WM columns—particularly within the fasciculus gracilis—between severe SCI and sham animals showed decreasing trend along the spinal cord rostral to the injury site at T8 vertebral level (Table 2). This is in line with histological^2,51^ and imaging studies,^10,45,52–55^ which have located the most severe damage in proximity to the primary injury site. A recent study demonstrated a similar neurodegenerative gradient within the corticospinal tract in the upper cervical cord of tetraplegic SCI patients.^56^ The neurodegenerative gradient can be partly attributed to the anatomical organization of the spinal cord. The injury at T8 vertebral level does not directly affect the axonal fibers entering or exiting the spinal cord above T8; therefore, the proportion of axons unaffected by the injury increases in more rostral locations.

Although not statistically significant, lower FA values (−7% to −10% compared with sham) were also detected in the dorsal columns of the mild and moderate SCI groups, rostral to the injury site (T1–T6 spinal levels), with even larger differences observed within the fasciculus gracilis (Table 2). We note that DTI metrics may not have reached their endpoint due to the presence of cellular debris at 90 dpi, as discussed further in the “Limitations” section. Nonetheless, the effect sizes observed in the mild and moderate SCI groups in the dorsal columns, a few spinal levels rostral to the injury, are similar to those previously reported in chronic incomplete tetraplegic SCI patients. In that study, patients exhibited 10% lower FA in the dorsal columns up to 7 spinal level rostral to the injury when compared with healthy controls.^27^

This study corroborates previous reports that DTI is sensitive to neurodegeneration following SCI. Decreased FA has been consistently reported at and near the injury site,^9,10,45,53,55^ but also rostrally and caudally.^16,53^ Due to dropouts, we could not statistically evaluate the FA for the severe SCI animals at the upper cervical segment, preventing us from performing a direct comparison with the histological findings, which were obtained at C2–C5. However, the mild and moderate SCI groups, where FA values are available for C3–C5, showed 7–8% lower FA values in the fasciculus gracilis compared with sham animals (Table 2). The fact that we observed reduced FA in areas of axonal degeneration supports the notion that axonal loss, among other mechanisms such as demyelination, reduces diffusion anisotropy by breaking the axonal cytoarchitecture.^8,45^ We did not observe significant differences in MD, AD, or RD between sham and SCI animals at any spinal segment, except for the severe SCI group, which showed lower AD in the dorsal columns at T4–T6 compared with the sham group. This is line with the results of a previous *in vivo* study with a rat contusion SCI model that found no significant effect of injury in MD, AD, and RD when computed by the DTI model, but did find significant effects in MD and AD when obtained by the free water elimination diffusion model.^16^ In contrast, other *ex vivo* MRI studies reported MD, AD, and RD decrease rostral to the lesion epicenter in rat contusion SCI models.^52,55^

### Remote gray matter degeneration rostral to injury: insights from imaging

A contusion SCI was previously shown to reduce the number of neurons in the dorsal, intermediate, and ventral GM laminae at and around the injury epicenter.^37,57–59^ Interestingly, we found signs of GM atrophy even in the upper cervical cord (Fig. 6A), suggesting that the longitudinal extent of neural loss is comparable or even larger than 15–25 mm reported in a thoracic contusion model,^60^ and much larger than 6 mm reported in cervical contusion models.^61,62^ These findings are also in line with the GM atrophy observed remote to the lesion in the cervical^63^ and lumbar cord^64^ of SCI patients. The extent of GM atrophy also showed a decreasing trend with increasing distance to the injury site (Table 1). Pathophysiological mechanisms for trans-synaptic GM degeneration include apoptosis and autophagy of the long propriospinal neurons,^65^ type of interneurons that are spread across the dorsal and ventral GM horns, and impaired metabolism resulting from damage to the vasculature^37,66^

### Relationship of histological measures and MRI metrics to locomotion

SMI-32 labeling strongly correlated with BBB scores at all time points, suggesting that the number of degenerating axons is a good descriptor of the locomotor function (Fig. 8A). As functional locomotion relies on feedback from muscle afferents, we argue that disruption of the proprioceptive system at the periphery of the medio- and ventrolateral columns may very likely contribute to the observed locomotor impairments. Axonal degeneration was found to be more related to injury at earlier time points, which is probably because many axons have already been removed by the inflammatory response at later time points. Previous studies found BBB scores to correlate with the amount of spared tissue^67^ and FA at the lesion epicenter,^53^ and with AD and MD at remote rostral locations.^16^ Here, we add to this by showing that local tissue volume and cross-sectional area of dorsal columns and GM at remote rostral segments also correlate with locomotor function in the chronic stage (Figs. 6B and 8B).

### Limitations

We used separate cohorts of animals for MRI and histology, and therefore could not make any direct comparisons between the two modalities. A limitation of histology is the difficulty to control for the SMI-32+ labeling variability between samples. Subtle SCI-related changes could be masked by slight variations in tissue preparation and labeling. Furthermore, MRI was limited to *ex vivo* measurements at 90 dpi. We note that the presence of SMI-32+ debris, indicative of axonal fragments, along with myelin fragments, is likely to impact the DTI measurements at 90 dpi. A previous study using a rat dorsal root axotomy model found that axonal degeneration coincided with a reduction in AD, which stabilized beyond 3 dpi, while a gradual increase in RD between 3 and 30 dpi correlated with the progressive clearance of myelin.^34^ Extrapolating to 90 dpi, we anticipate that RD has not yet reached its maximum (and correspondingly, FA has not yet reached its minimum). The ongoing clearance of cellular debris may contribute to a further increase in RD and decrease in FA. Additional MRI scans at earlier and later time points or longitudinal assessments may be valuable to further understand the noninvasive signatures and evolution of remote degeneration.

Other limitations are related to the BBB scores as a measure of locomotor function. BBB scores are subject to intra-rater, inter-rater, and test-retest variability, and are not sensitive to functional changes in mild spinal cord injuries.^68^ Furthermore, accurate BBB scoring may be confounded by pain, inflammation, and incisions at the early stages of injury.^69^

While Experiment 1 used male rats and Experiments 2 and 3 used females, this does not pose a concern, as we did not directly compare results between sexes in our analyses. Instead, we replicated the histological findings in two independent experiments using different sexes, demonstrating the reproducibility of the observed phenomenon. Moreover, although a previous study reported sex-based differences, with female rats showing improved locomotor recovery and greater preservation of white and gray matter within the injured spinal cord segment following traumatic SCI,^70^ these effects were smaller than those associated with injury severity (Experiments 2 and 3).

DTI is an imperfect model, and the interpretations and conclusions must be supported by the behavior of the underlying model.^71^ DTI metrics are sensitive but not specific to pathological processes. For example, fractional anisotropy can indicate both axonal and myelin loss but can also be affected by other physiological factors such as inflammation.^72^ A modification of DTI, the free water elimination technique reduced partial volume effects at the cord boundary in the rat spinal cord and showed higher correlation to BBB scores.^16^

## Conclusions

Using a combined histological and imaging approach, we demonstrated in a rat thoracic contusion SCI model that remote axonal degeneration occurs in the cervical cord as early as 2 dpi and persists over 90 dpi, possibly much longer. Clinically established structural and diffusion MRI sequences proved sensitive to capture above-lesion atrophic and degenerative processes. These findings help understand the mechanisms underlying imaging-based clinical observations of remote secondary degeneration in SCI patients.

## Transparency, Rigor, and Reproducibility Statement

This study was not formally registered, nor was the analysis plan preregistered. All imaging data were collected using the same 9.4T Bruker BioSpec scanner and processed with the same pipeline. Image processing was automatic and did not require manual intervention. BBB scores were assigned to animals by two raters blinded to the data. Data and analytic code will be available upon reasonable request from the corresponding author. The key findings from our study (i.e., neurodegeneration remote to focal injury) were reproduced in two separate experiments, as reported in this article (Experiments 1 and 2), conducted at different centers by independent investigators. In Experiment 1, the sample size was 9 (5 SCI and 4 sham rats), while in Experiment 2, the 72 rats were evenly distributed across four injury groups and three time points (*n =* 6 for each category). These sample sizes were well powered given the high sensitivity of SMI-32 immunohistochemistry to degenerative changes. In Experiment 3, the sample size was 27, distributed across four injury severity groups. Due to low quality, one sham and one severe SCI specimen were excluded from the analysis of cross-sectional areas, and two sham and two severe SCI specimens from the analysis of DTI metrics. The cervical segment was not available in an additional severe SCI specimen. When assessing pairwise group differences in the MRI metrics, we used Bonferroni correction to control for Type I errors.

## Abbreviations Used

AD: Axial diffusivity
ANOVA: Analysis of variance
BBB: Basso, Beattie, and Bresnahan
dpi: Days post-injury
DTI: Diffusion tensor imaging
FA: Fractional anisotropy
FOV: Field of view
FWE: Family-wise error
GM: Gray matter
HPF: High-power field
MD: Mean diffusivity
MRI: Magnetic resonance imaging
RARE-VTR: Rapid Acquisition with Relaxation Enhancement at Variable Repetition Time
RD: Radial diffusivity
ROI: Region of interest
RT: Room temperature
SCI: Spinal cord injury
TE: Echo time
TR: Repetition time
WM: White Matter
